# The Interplay Between Periodontitis and Atrial Fibrillation: Inflammation as a Common Pathophysiological Bridge

**DOI:** 10.3390/ijms27073082

**Published:** 2026-03-28

**Authors:** Francesco Caprino, Andrea Filardo, Jessica Bria, Isabella Coscarella, Amerigo Giudice, Emanuela Chiarella, Anna Di Vito

**Affiliations:** 1 Department of Experimental and Clinical Medicine, University Magna Græcia, 88100 Catanzaro, Italy; f.caprino@unicz.it; 2 Department of Medical and Surgical Sciences, University Magna Græcia, 88100 Catanzaro, Italy; filardo@unicz.it (A.F.); jessica.bria@studenti.unicz.it (J.B.); isabella.coscarella@studenti.unicz.it (I.C.); emanuelachiarella@unicz.it (E.C.); 3 Department of Health Science, School of Dentistry, University Magna Græcia, 88100 Catanzaro, Italy; a.giudice@unicz.it

**Keywords:** periodontitis, atrial fibrillation, microbial dysbiosis, chronic inflammation, immune response, inflammasome

## Abstract

Periodontitis (PD) and atrial fibrillation (AF) are two prevalent chronic conditions with substantial public health burdens worldwide. While traditionally studied separately, increasing evidence reveals a complex interplay between PD and AF, mediated primarily by shared inflammatory and immune mechanisms. Chronic periodontal inflammation can trigger systemic immune activation, leading to atrial structural remodeling, fibrosis, and electrical disturbances that predispose individuals to AF. Observational and longitudinal studies consistently demonstrate a higher incidence and recurrence of AF in patients with moderate to severe PD, independent of established cardiovascular risk factors. Key periodontal pathogens, especially *Porphyromonas gingivalis*, and altered immune cell profiles are implicated in this association, further supported by genetic analyses revealing common molecular pathways. Mechanistic insights from experimental models highlight the role of inflammation-related atrial fibrosis and immune dysregulation as critical drivers linking oral disease to arrhythmogenesis. Additionally, better oral hygiene practices and periodontal treatment have been associated with a reduced risk of AF, suggesting modifiable intervention potential. This review synthesizes current clinical, epidemiological, molecular, and experimental evidence to elucidate the PD–AF relationship, emphasizing periodontal health as a promising target in cardiovascular disease prevention strategies.

## 1. Introduction

Atrial fibrillation (AF), the most prevalent sustained cardiac arrhythmia, affects over 33 million people globally and contributes significantly to stroke, heart failure, and cardiovascular mortality [1]. Concurrently, periodontitis (PD) remains one of the most widespread chronic inflammatory diseases, impacting nearly half of the adult population, with severe forms affecting up to 10% [2,3]. Though seemingly unrelated, a growing body of evidence points to an unexpected and clinically meaningful intersection between the two conditions.

Chronic PD, driven by microbial dysbiosis and persistent local inflammation, initiates a cascade of systemic immune activation and proinflammatory cytokine release, systemic responses that can reach the heart, where they contribute to atrial structural remodeling, fibrosis, and electrophysiological disturbances that predispose the patient to AF [4,5,6]. Moreover, immune and genetic analyses have identified shared pathways between the two conditions, such as overexpression of the Regulator of G-protein Signaling 1 (RGS1) gene and T-cell dysregulation, further supporting a common immunoinflammatory substrate [6].

Recent studies have proposed a mechanistic bridge between PD and AF, outlining how local oral inflammation can provoke systemic responses that lead to cardiac arrhythmias; this biological rationale is corroborated by a range of clinical, epidemiological, and experimental findings. Patients with moderate to severe PD exhibit a significantly higher risk of developing AF, independent of conventional cardiovascular risk factors [3,4,7,8,9,10,11,12]. Additionally, poor oral hygiene and tooth loss—often surrogates of advanced PD—are consistently associated with greater atrial fibrosis and thrombus formation.

This review synthesizes current evidence linking PD and AF across multiple levels of inquiry, from mechanistic biology to real-world population studies, aiming to explore shared immunopathological pathways, highlight clinical and genetic correlations, and evaluate whether periodontal health could serve as a novel, modifiable factor in cardiovascular risk prevention. By integrating oral health into cardiovascular care, clinicians and researchers may uncover new opportunities for interdisciplinary management and disease prevention.

## 2. Materials and Methods

This narrative review provides an overview of the interplay between PD and AF, focusing on inflammation as a common pathophysiological bridge. A comprehensive literature search was conducted using PubMed, Scopus, and Web of Science, with publications included up to December 2025. Keywords used for the search included “periodontitis and atrial fibrillation”, “inflammation and periodontitis”, “inflammation and atrial fibrillation”, “immune system and periodontitis”, and “immune system and atrial fibrillation”. The inclusion criteria were as follows: prospective or retrospective studies, including randomized controlled trials, cohort studies, case–control studies, cross-sectional studies, or reviews on the relationship between PD and AF; PD and oral care, such as dental cleaning and frequent tooth brushing; and new-onset or recurrent AF as outcome indicators. Studies were selected based on relevance to the topic. Data were synthesized descriptively, with a focus on the key themes identified in the review.

## 3. Relationship Between Periodontitis and Atrial Fibrillation

The interplay between unbalanced oral microbiota and the host’s chronic immune response in advanced PD creates a persistent, systemic, low-grade inflammatory state that is a critical driver for numerous systemic diseases; among them, cardiovascular diseases are the leading cause of death worldwide and can result from chronic PD through two mechanisms: a direct pathway, through the invasion of pathogenic bacteria, and an indirect pathway, through the release of inflammatory cytokines into circulation [13].

Recent preclinical and observational cohort studies have implicated dysbiosis as a contributor to AF. AF is the most common sustained cardiac arrhythmia worldwide, with a lifetime risk of approximately one in three; its incidence continues to rise in parallel with population aging and the increasing burden of established cardiovascular risk factors, including hypertension, diabetes mellitus, obesity, heart failure, and valvular heart disease, and it is associated with increased risks of ischemic stroke, heart failure, and all-cause mortality, making its clinical burden substantial [14,15]. From a clinical point of view, AF is associated with an approximately five-fold increase in ischemic stroke risk and a three-fold increase in heart failure incidence, underlining the need for long-term anticoagulation and integrated multidisciplinary care pathways [16]. Importantly, the loss of coordinated atrial systole during AF abolishes the physiological “atrial kick,” leading to reduced ventricular filling, decreased cardiac output, and blood stasis—particularly within the left atrial appendage—thereby markedly increasing the risk of thrombus formation and cardioembolic events [16]. Clinically, this hemodynamic impairment contributes not only to thromboembolic risk, but also to symptoms such as palpitations, exertional intolerance, hypotension, dyspnea, dizziness, and worsening heart failure, especially in patients with diastolic dysfunction and persistent or permanent AF phenotypes, due to which patients with AF also have decreased quality of life [14]. From a clinical risk stratification perspective, the loss of effective atrial systole, and the consequent blood stasis within the left atrial appendage, represents a central pathophysiological mechanism underlying thromboembolic risk in AF, directly informing anticoagulation decisions based on CHA_2_DS_2_-VASc scoring and the high prevalence of LAAT (left atrial appendage thrombus) in sustained AF forms [16,17]. Clinical studies have linked markers of periodontal inflammation—such as Periodontal Inflamed Surface Area (PISA), Bleeding on Probing (BOP), and Probing Pocket Depth (PPD)—to atrial fibrosis and thrombus formation [5]. Histological evaluation of left atrial appendage specimens from AF patients demonstrates that PISA, BOP, and PPD correlate positively with the degree of atrial fibrosis, reinforcing that chronic periodontal inflammation associates with structural cardiac remodeling [5]. Accordingly, patients with non-paroxysmal AF (including persistent AF, long-standing persistent AF, and permanent AF), or left atrial appendage thrombus (LAAT), show significantly higher PISA values and more advanced periodontal disease, supporting a direct association between periodontal inflammation and adverse atrial remodeling [5]. Clinically, progression from paroxysmal to persistent and permanent AF is associated with worsening symptom severity, reduced efficacy of rhythm control strategies, higher recurrence rates after catheter ablation, and increased healthcare utilization [17]. In addition, observational evidence suggests that severe PD is associated with an elevated risk of developing AF in the general population [18]. Clinically, patients with concomitant periodontal disease and AF also appear to have worse outcomes after rhythm control interventions. For example, PD is independently associated with increased arrhythmia recurrence after catheter ablation for paroxysmal AF, indicating that periodontal status may influence procedural success rates [19]. Beyond arrhythmia recurrence, periodontal disease may contribute to systemic thromboembolic risk. AF patients with poor periodontal health exhibit higher inflammatory burdens and may face elevated thrombotic and stroke risk compared to those without PD [11]. Together, these clinical data suggest that periodontal disease not only increases AF incidence, but also worsens AF progression, recurrence risk, and associated complications, underscoring the potential importance of integrating periodontal assessment and management into comprehensive AF care pathways.

## 4. Periodontitis and Atrial Fibrillation: Insights from Clinical, Genetic, and Population-Based Studies

An expanding body of evidence supports a significant association between PD and AF, suggesting that these two highly prevalent conditions may share common pathophysiological mechanisms and risk factors. Both disorders increase in prevalence with age and are influenced by chronic systemic inflammation, immune dysregulation, and adverse lifestyle behaviors. While epidemiological and population-based studies consistently reveal a link between PD and AF, moving from association to causation requires greater mechanistic understanding. The following section explores the clinical, genetic, and epidemiological evidence of association between PD and AF (Table 1).

Clinical investigations have played a key role in delineating the relationship between oral health and cardiac pathology. Struppek et al. observed that the severity of both PD and AF increases with age, with initial correlations identified between severe PD and AF [3]. However, after adjusting for confounding variables, only the plaque index remained significantly associated with AF (odds ratio (OR): 1.22, 95% confidence interval (CI): 1.1–1.35, *p* < 0.001). Notably, individuals who practiced better oral hygiene—specifically brushing twice daily—showed a lower prevalence of AF, suggesting a protective role of routine dental care. Interestingly, systemic inflammatory markers, such as IL-6 and CRP, did not mediate this association, implying that localized or chronic oral inflammation might exert a more direct influence on AF risk. Further mechanistic support comes from Goette, who proposed that chronic periodontal inflammation may promote atrial fibrosis through the action of inflammatory cytokines and bacterial byproducts [20].

Building on these clinical insights, Miyauchi et al. examined periodontal status across different AF subtypes [5]. In a cohort of 76 AF patients undergoing left atrial appendage excision, the severity of atrial fibrosis correlated with both the extent of PD and the duration of AF; their study found that patients with non-paroxysmal AF had significantly higher PISA values compared to those with paroxysmal AF. Moreover, patients with left atrial appendage thrombus (LAAT) exhibited even greater periodontal inflammation, fewer remaining teeth, and a higher overall periodontal disease burden. The associations between PISA, BOP, and PPD ≥ 4 mm and atrial fibrosis remained significant after adjusting for cardiovascular comorbidities, reinforcing the idea that PD may influence not only AF onset, but also its progression and associated thromboembolic complications, through atrial remodeling.

Xiang et al., in their bioinformatics analysis, identified genetic overlaps between PD and AF [6]. Through transcriptomic and immune cell profiling, they discovered 21 genes shared between the two conditions, with RGS1 emerging as a key regulatory node. Experimental rat models of PD confirmed these findings, showing increased susceptibility to AF, enhanced atrial fibrosis, and upregulated RGS1 expression in cardiac tissue, results which suggest that a shared immunogenetic pathway may underlie the PD–AF connection, extending beyond traditional inflammatory markers.

Complementing clinical and genetic findings, robust epidemiological studies further support this link. Sen et al., in a 17-year cohort study, demonstrated that moderate to severe PD independently predicted AF incidence (severe PD; HR, 1.31, 95% CI, 1.06–1.62) [11]. Mediation analysis revealed that AF partly explained the elevated stroke risk in PD patients. Moreover, individuals receiving regular dental care had a lower incidence of AF (HR, 0.88, 95% CI, 0.78–0.99), underscoring the preventive potential of maintaining good periodontal health.

Large-scale population-based studies further consolidate this association. Chen et al., in a study involving over 390,000 matched individuals, found a significantly higher AF incidence in those with PD (2.07% vs. 1.57%) [12], an association that remained robust across demographic and clinical subgroups after adjustment (HR 1.31). Hsu et al., in a study involving 282560 PD and non-periodontitis patients showed that chronic PD is associated with AF (OR, 1.39, 95% CI, 1.30–1.48) [21]. Park et al., in a 14.3-year longitudinal analysis, found that individuals with chronic or newly diagnosed PD had significantly higher risk (HR, 1.04, 95% CI, 1.01–1.08) of developing AF compared to PD-free individuals [9]. Notably, those who recovered from PD showed a risk profile similar to that of individuals without PD, suggesting that effective periodontal management may reverse AF susceptibility (HR, 0.97, 95% CI, 0.94–0.99).

Taken together, clinical, genetic, and epidemiological evidence appears to provide convincing support for the hypothesis that PD may contribute to both the onset and progression of AF. The interplay among chronic oral inflammation, immune dysregulation, atrial remodeling, and genetic susceptibility outlines a biologically plausible framework linking these two conditions. Mechanistic insights highlight the roles of shared immune signatures, proinflammatory cytokines, and microbial triggers as potential contributors to both disease processes. Importantly, the consistent association between good oral hygiene and reduced AF risk underscores the possibility that periodontal health may serve as a modifiable and clinically actionable factor in cardiovascular disease prevention, emerging insights which not only strengthen the argument for causality but also pave the way for future integrated prevention and management strategies.

**Table 1:** Summary of key clinical, genetic, and epidemiological studies investigating the association between PD and AF. The table outlines study designs, main findings, OR, HR, CI, and relevant references, highlighting evidence that links PD severity, periodontal treatment, and oral hygiene with AF risk and progression.

Design & Population	Periodontal Parameters & Methodology	Key Statistical Outcomes (95% CI)	Adjustment Factors (Confounders)	Mechanistic Insights & Clinical Implications	Study (Ref.)
Cross-sectional study. Age-varied adults from the Hamburg City Health Study.	Assessment of PD severity and dental plaque index (PI); AF prevalence evaluated.	PD severity associated with AF: OR 1.22 (1.10–1.35)	Age and demographic variables (as per population-based cohort adjustment)	Oral hygiene represents a modifiable factor. Systemic inflammatory markers (IL-6, CRP) did not mediate the association, suggesting alternative inflammatory or structural pathways.	[3]
Cross-sectional Study. Cohort of 76 AF patients undergoing LAA excision (histological study)	Measurement of PISA (Periodontal Inflamed Surface Area) and histological atrial fibrosis.	No HR/OR Significant correlation between higher PISA scores and increased atrial fibrosis levels.	Adjusted for AF duration and type (paroxysmal/persistent), BMI, mitral valve regurgitation, and CHADS2-VASc score.	Direct biological link: oral inflammation severity correlates with structural heart damage.	[5]
Genetic and transcriptomic bioinformatic analysis. Rat model validation.	Identification of shared genes between PD and AF; immune-cell infiltration analysis; experimental PD induction in rats.	No HR/OR 21 common genes identified; *RGS1* associated with immune cell infiltration and fibrosis.	Not applicable (bioinformatic and experimental model).	Not applicable (bioinformatic and experimental model).	[6]
Longitudinal Cohort Study. 14.3 years of follow-up to evaluate the long-term impact of PD	Comparison between chronic PD, newly diagnosed PD, and recovered (treated) PD patients.	Chronic/New PD and AF: HR 1.04 (1.01–1.08). Recovered PD and AF: HR 0.97 (0.94–0.99).	Time-dependent Cox models adjusted for BMI, alcohol, smoking, and blood pressure.	Treating PD to a “recovered” state can reverse the excess risk of developing AF.	[9]
National Cohort Study. Large-scale population analysis (N > 6700); patients enrolled in Dental Atherosclerosis Risk in Communities Study (DARIC).	Participants were grouped into periodontal health, mild PD, moderate PD, and severe PD groups.	PD severity was associated with AF: HR 1.31, (1.06–1.62). Regular dental care and AF: HR, 0.88, (0.78–0.99).	Adjusted for age ≥ 60, race, sex, hypertension, diabetes, LDL ≥ 100, obesity, smoking, alcohol use, preexisting heart conditions, and education level.	PD is associated with AF. Oral hygiene represents a modifiable factor.	[11]
National Cohort Study. Large-scale population analysis (N > 390,000) using medical databases.	Patients with new PD diagnosis vs. healthy controls. Impact of regular scaling (≥1/year).	PD incidence on AF: HR 1.31 (1.25–1.36). Scaling (≥1/yr for 3 yrs): HR 0.67 (0.52–0.86).	Matched for age, sex, income, and various cardiovascular comorbidities (CCI).	PD is associated with AF. Regular professional scaling acts as a primary prevention tool against AF.	[12]
National Cohort Study. Large-scale population analysis (N = 565,120) using medical databases.	Patients with PD diagnosis vs. healthy controls.	Chronic PD and AF: OR 1.39 (1.30–1.48).	Confounding factors not assessed	PD is associated with AF.	[21]

## 5. Molecular Mechanisms Linking Periodontitis to Atrial Fibrillation

PD is a chronic inflammatory condition triggered by microbial dysbiosis within dental plaque accumulated in the periodontal pocket. This localized infection represents the starting point of a complex and multifaceted pathogenic cascade that extends far beyond the oral cavity. The ulcerated epithelium of the periodontal pocket acts as a gateway, where the local inflammatory response and microbial imbalance facilitate the periodic translocation of periodontal pathogens—such as *Porphyromonas gingivalis*—and their virulence factors into the systemic circulation [22].

Once in the bloodstream, these agents trigger a state of persistent systemic inflammation and chronic immune activation. This condition is clinically evidenced by elevated levels of circulating pro-inflammatory cytokines, including IL-6, TNF-α, and IL-1β, and significant immune alterations, such as the expansion of memory CD4+ T cells and the upregulation of specific genes involved in immune cell trafficking and localization.

The persistence of systemic inflammatory burdens can eventually extend to cardiac tissues, where it promotes atrial structural remodeling and fibrosis acting as determinants of atrial ectopy and conduction abnormalities. Several molecular mechanisms are involved in these processes, such as altered ion channel expression, Ca^2+^ handling abnormalities, changes in connexins expression, activation of atrial fibroblasts, and excessive deposition of extracellular matrix (ECM) components. ECM remodeling increases atrial stiffness and creates significant electrical disturbances, disrupting normal conduction pathways and reducing the threshold for arrhythmia [23]. Specifically, the fibrotic tissue interferes with the seamless propagation of electrical impulses, creating an anatomical environment favorable to re-entry circuits.

**Figure 1:**
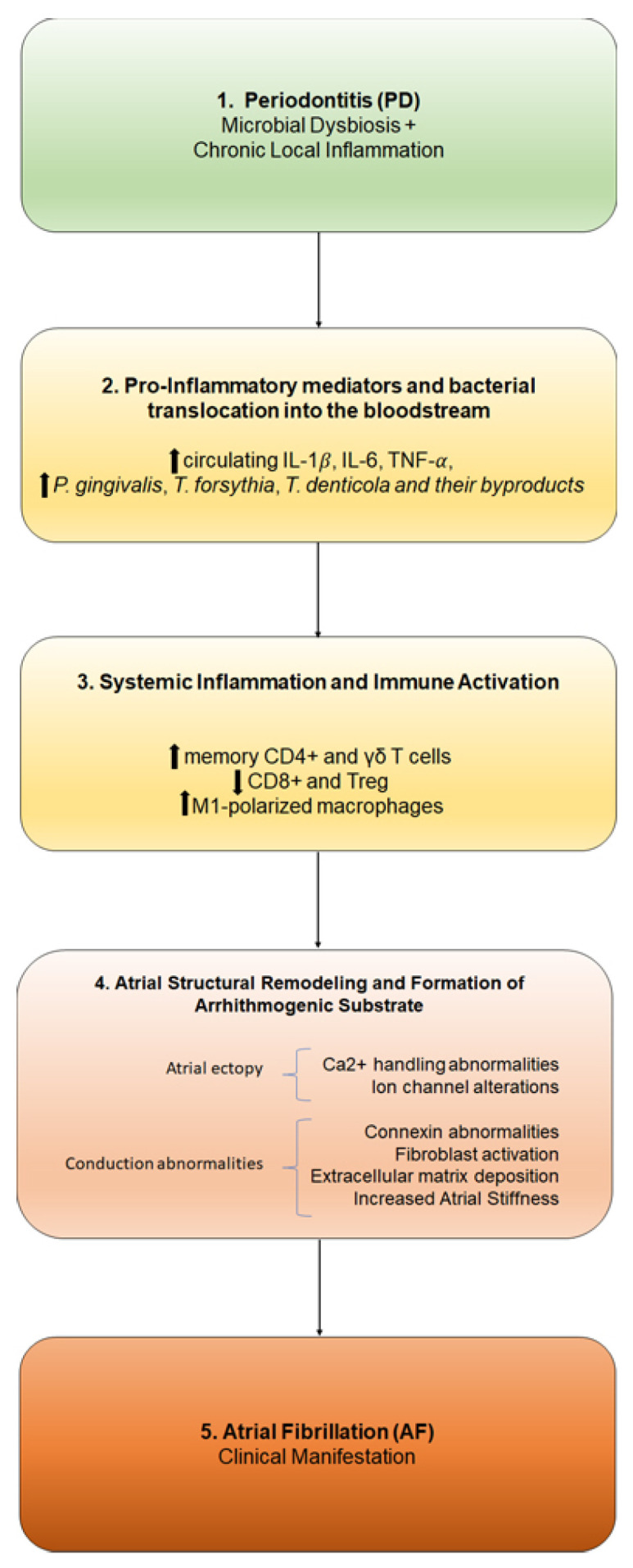
Proposed mechanisms of atrial fibrillation in periodontitis.

In this context, AF may arise as a clinical manifestation of this progressive pathophysiological deterioration, potentially representing a long-term downstream consequence of chronic, untreated PD (Figure 1). This mechanistic link underscores the broader systemic implications of oral disease, suggesting that the “oral–cardiac axis” is a critical pathway. Consequently, these findings highlight the necessity for integrated care strategies and intensified interdisciplinary research to mitigate the cardiovascular risks associated with periodontal infection.

The biological connection between PD and AF is complex, involving intertwined microbial factors, pathways of chronic inflammation, immune dysregulation, and morpho-functional remodeling of cardiac tissue. Understanding these mechanisms is essential to elucidate how a localized oral disease might influence cardiac electrophysiology and arrhythmogenesis. Below, we organize current knowledge of molecular mechanisms into three interrelated domains.

### 5.1. Microbial Dysbiosis and Pathogens Translocation as Triggers of Systemic Inflammation

The biofilm is a surfaced-attached microbial community surrounded by an ECM of biological macromolecules that covers tooth surfaces. Currently, only a fraction of the approximately seven hundred bacterial species that coexist in the oral cavity has been characterized [24]. When the disturbance of the normal ecological balance of the oral biofilm occurs, a condition known as dysbiosis, pathogenic bacteria overgrow and contribute to periodontal disruption through synergistic mechanisms [25,26]. Generally, microbial community imbalance is more evident in subgingival plaque samples, followed by saliva, and tongue biofilm [26].

PD is categorized into stages I–IV and grades A, B, and C, where stages indicate disease severity and management complexity, while grades indicate progression rate [27]. Interestingly, oral biofilm showed specific species combinations that differ in the different stage of PD [26]. In this context, in 1998, Socransky identified five bacterial complexes associated with periodontal disease: purple, green, yellow, orange, and red. Among these, the red complex is closely related to the most severe forms of PD or chronic PD, occurring in elderly population [28]. This group includes highly pathogenic bacteria which are *Tannerella forsythia*, *Porphyromonas gingivalis*, and *Treponema denticola*. The orange complex, considered the precursor of the red complex in the process of microbial colonization, includes *Fusobacterium nucleatum*, *Prevotella intermedia*, *Prevotella nigrescens*, *Peptostreptococcus micros*, *Streptococcus constellatus*, *Eubacterium nodatum*, *Campylobacter showae*, *Campylobacter gracilis*, and *Campylobacter rectus*. The yellow complex includes several Streptococcus species, while the green complex includes three Capnocytophaga species, *Campylobacter concisus*, *Eikenella corrodens*, and *Aggregatibacter actinomycetemcomitans*. Finally, the purple complex includes *Veillonella parvula* and *Actinomyces odontolyticus*.

More recently, in their elegant research, Oliveira Fernandes et al. revisited Socransky’s Complexes with the specific aim of including newly discovered bacteria from individuals with periodontal/peri-implant diseases [29]. They organized bacteria into six group or Clusters associated with different healthy or pathological conditions, namely health, gingivitis, peri-implant mucositis, PD, peri-implantitis, and necrotizing and molar–incisor pattern PD [29]. In this updated version, the seven most significant bacteria in PD were all gram-negative: *Porphyromonas gingivalis*, *Aggregatibacter actinomycetemcomitans*, *Tannarela forsythia*, *Prevotella intermedia*, *Fusobacterium nucleatum*, *Treponema denticola*, and *Campylobacter rectus*. Interestingly, Socransky’s red complex associated with chronic PD (*P. gingivalis*, *T. forsythia*, *T. denticola*) was confirmed by Oliveira Fernandes et al., which included these bacteria in the Cluster indicated by a red triangle with the addition of *Aggregatibacter actinomycetemcomitans*. The inclusion of *Aggregatibacter actinomycetemcomitans* in the red triangle was due to its presence in all the Clusters, especially in necrotizing and molar–incisor pattern PD.

There is a noteworthy correlation between a different form of periodontal disease, referred to as aggressive PD, which generally affects younger patients and shows a more rapid progression, and the presence of *Aggregatibacter actinomycetemcomitans* [30,31].

Interestingly, while the presence of *Fusobacterium nucleatum* alone does not determine periodontal inflammation, its increased abundance has been associated with the onset of PD-specific bacteria accumulation, indicating a role in the development of PD [32,33].

**Figure 2:**
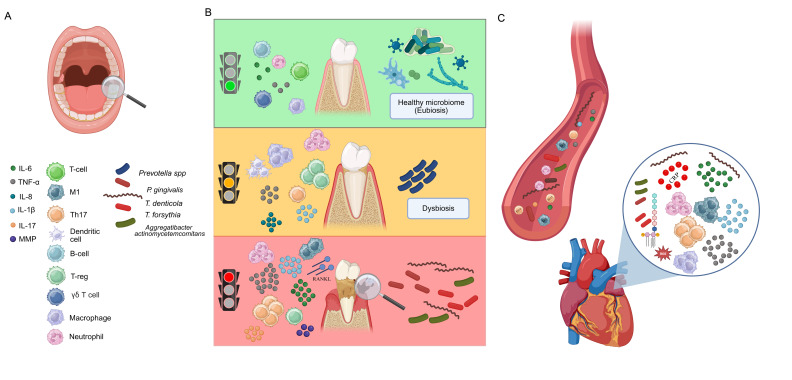
Oral dysbiosis and systemic inflammation. Oral pathogens in dental plaque induce a local immune response and inflammatory activation, both recognized as triggers of gingivitis (**A**,**B**). Without proper treatment, gingivitis can evolve into periodontitis, causing gum recession, pocket formation, and bone and tissues disruption (**B**). Bacteria and their byproducts, as well as inflammatory mediators, enter the bloodstream leading to systemic inflammation that can reach the heart (**C**) (created using https://BioRender.com).

From a clinical point of view, after microbial imbalance, both host immunity activation and sustained inflammation configured as the initiators of gingivitis (Figure 2). This condition is reversible with early detection, professional care, and consistent oral hygiene practices. However, if not properly managed, gingivitis can progress to PD, which can eventually lead to the entry of bacteria or their byproducts into the bloodstream [34]. These specific periodontal pathogens may act as remote biological triggers of atrial remodeling, becoming key factors in the pathogenesis of AF. Accordingly, elevated serum IgG antibody titers against *Porphyromonas gingivalis* type IV have been independently associated with an increased risk of AF recurrence after catheter ablation in both paroxysmal and persistent forms [35]. Similarly, a higher prevalence of positive serum titers of antibodies against *Porphyromonas gingivalis* type III and type V was reported in patients with AF when compared to those without AF, within a cohort of acute stroke patients [36].

The potential for systemic translocation of oral pathogens is widely recognized and most evidence arises from identifying DNA from periodontal bacteria in distant sites [37]. What are the mechanisms that determine bacterial dissemination into the systemic circulation? Periodontal tissues are highly vascularized, and the breakage of blood vessels may occur as consequences of either gingival tissue trauma or dental procedures, allowing oral pathogens and their components to directly enter the bloodstream, causing bacteremia (Figure 2) [38].

Additionally, the local immune response to dysbiosis could strongly contribute to connective tissue disruption and bacteria dissemination in the blood. Indeed, during PD, neutrophils and monocytes undergo epigenetic and metabolic reprogramming, Th17/Treg homeostasis is deregulated, and macrophages acquire a pro-inflammatory phenotype M1, accounting for heightened inflammatory state [39,40]. This process, indicated as “maladaptive trained immunity”, is responsible for the sustained release of pro-inflammatory mediators (IL-1β, IL-6, IL-8, TNF-α, TGF-β, IL-17, IL-18, IL-23, PGE2, etc.) which contribute to tissue disruption and could also spill into blood, contributing to systemic inflammation [41]. Altogether, proinflammatory mediators derived from immune cells, circulating bacteria, and their immunogenic products, such as bacterial DNA and endotoxins (proteases, LPS, etc.), trigger systemic inflammation (Figure 2). These events could be further intensified by the exacerbation of autoimmunity response against biomolecules expressed in the heart, such as HSP60/65 and citrullinated cardiac proteins, as a consequence of molecular mimicry events [42,43,44].

Interestingly, maladaptive immunity in response to PD was also observed in bone marrow, where the presence of trained myeloid progenitors has been associated with increased local and systemic inflammation [40].

Notably, while cardiovascular implications of pro-inflammatory interleukins derived from PD are increasingly clear, the causal relationships between specific inflammatory mediators and AF are still poorly understood. As mentioned above, both host immunity activation and sustained inflammation could contribute to atrial tissue damage through different mechanisms, including overproduction of ROS in heart tissues, altered platelet activation, and activation of local immune-inflammatory processes [45]. Oxidative stress contributes to the development and maintenance of AF by electrical remodeling, Ca^2+^ handling abnormalities, structural remodeling, and changes in the autonomic nervous system (reviewed in [46]). Furthermore, oxidative stress in platelets may lead to their hyperactivation and excessive clot formation, recognized as a hallmark of AF [47].

Cardiac immune-inflammatory processes consist of reciprocal cross-talks between cardiomyocytes, fibroblasts, and immune cells, resulting in the establishment of a reverberating proinflammatory and profibrotic circuit. In the next section, the mechanisms involved will be analyzed.

### 5.2. Inflammation and Immune Dysregulation Lead to the Formation of an Arrhythmogenic Substrate

**Figure 3:**
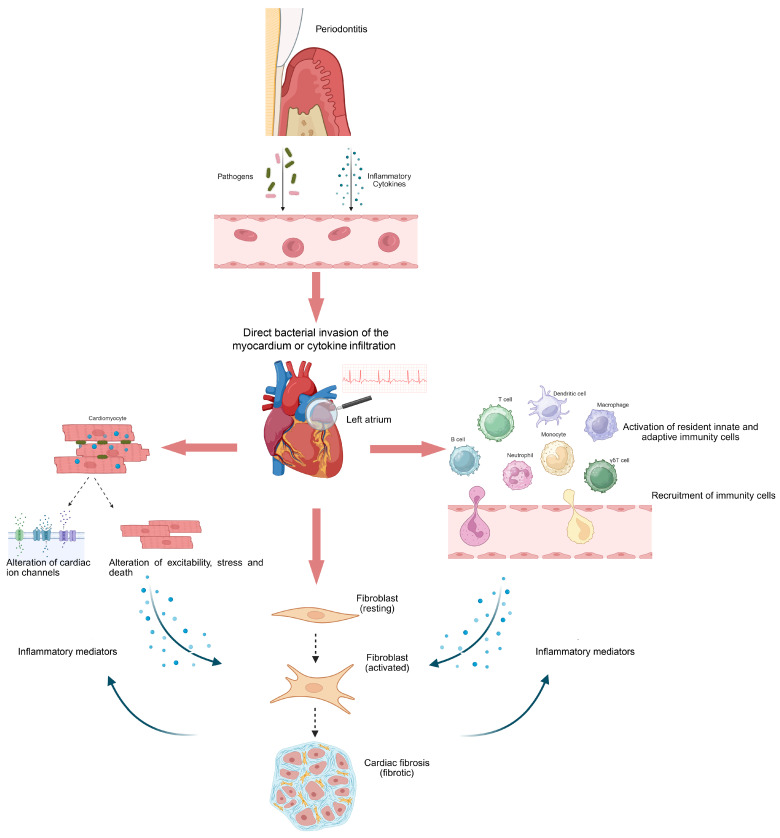
Role of periodontal pathogens and circulating inflammatory mediators in AF pathophysiology. Direct bacteria or cytokines infiltration of the heart contributes to the establishment of arrhythmogenic substrate by acting on fibroblasts, cardiomyocytes, and cardiac immune cells. The perpetuation of a proinflammatory arrhythmogenic substrate strongly depends on the interactions between cardiomyocytes, fibroblasts, and immune cells (created using https://BioRender.com).

Many studies have suggested the key role of immune-inflammatory processes in shaping arrhythmic substrates [48]. This evidence unravels the correlation between some medical conditions—such as chronic kidney disease, Chronic Obstructive Pulmonary Disease (COPD), diabetes, obesity, cancers, autoimmunity—and the risk of developing the structural and electrical issues that lead to AF. In all these cases, chronic inflammation stands as a potential link bridging the specific disease and AF. Although PD-dependent systemic inflammation might drive the development of AF through similar mechanisms, evidence of causality is still lacking. To date, it is only possible to show that both circulating bacteria and inflammatory mediators produced in PD enter the systemic circulation and reach the heart, where directly act on fibroblasts and cardiomyocytes promoting electrical and structural cardiac remodeling (Figure 3) [10,20,49,50].

Many inflammatory mediators have been associated with AF development, such as IL-1β, IL-2, IL-6, IL-8, TNF-α, TGF-β, C-reactive protein (CRP), cluster of differentiation 40 ligand (CD40L), endothelin-1, fibrinogen, matrix metalloproteinase (MMP)-9, monocyte chemoattractant protein (MCP)-1, myeloperoxidase (MPO), plasminogen activator inhibitor (PAI)-1, and serum amyloid A (SAA) [51,52]. Both MMPs and TIMPs have been associated with the occurrence of AF. Furthermore, MMP-9 was recognized as a fibrotic marker and associated with atrial structural remodeling and dilatation [53,54,55]. Atrial dilatation results from the cascade involving cytokines upregulation, MMPs activation, ECM remodeling and then fibrosis. Additional proinflammatory cytokines, including PGE2, immunoglobulin A, and immunoglobulin G are also upregulated in both PD and many cardiovascular diseases, suggesting the need for further investigation to clarify their involvement in AF [50].

These inflammatory mediators influence cardiomyocyte excitability and conduction by modulating ion channel expression and function. In the case of AF, ion channel abnormalities are associated with shortened effective refractory period and increased re-entry of cardiomyocytes and include mutations in genes encoding specific channel and functional alterations in Na^+^, K^+^, and Ca^2+^ channels. Specifically, an altered expression of potassium and hyperpolarization-activated cyclic nucleotide-gated (HCN) channels together with changes in Ca^2+^ handling were responsible for prolonged duration of action potential and enhanced repolarization heterogeneity in cardiomyocytes [56,57]. Consequently, there is an increased frequency of reentrant circuits. Interestingly, ion channel abnormalities were also reported in fibroblast [58]. It is clear that fibroblasts do not generate action potential and lack voltage-dependent ion channels. However, the upregulation of specific ion channels, including Transient Receptor Potential (TRP) and Na + channels, in fibroblasts has been associated to alterations in electrophysiology of cardiomyocytes [59]. Additionally, fibroblasts respond to inflammatory signals by secreting additional cytokines and matrix proteins, thus contributing to atrial fibrosis (Figure 3). On the other hand, circulating bacteria and their immunogenic byproducts activate both innate and adaptive immune responses that contribute to tissue remodeling. Macrophages, neutrophils, and dendritic cells promptly respond to bacterial signals by producing proinflammatory cytokines such as TNF-α, IL-1β, and IL-6, further promoting inflammation [60]. Similarly, T and B lymphocytes were recruited and contribute to either inflammation resolution or atrial remodeling (Figure 3) [61].

Immune dysregulation plays a key role in mediating the association between FA and PD. Recent transcriptomic and immune cell profiling studies have identified a shared immunogenetic signature between PD and AF. Notably, the RGS1 gene upregulated in both diseases, correlating with increased infiltration of activated memory CD4+ and γδ T cells and a concomitant reduction in CD8+ and Treg within atrial tissue [6]. This altered immune landscape contributes to persistent inflammation, fibrotic remodeling, and enhanced arrhythmogenicity. Experimental models further support this concept; rodents with induced PD demonstrated increased atrial fibrosis and heightened AF susceptibility alongside upregulated RGS1 expression [6]. These findings collectively suggest that systemic immune activation originating from periodontal inflammation can directly affect cardiac structure and function, promoting AF development.

Therefore, circulating cytokines and bacterial byproducts induce the activation of cardiomyocytes, fibroblasts, and innate and adaptive immunity, which establish mutual interactions mediated by direct contact, paracrine signaling, and the release of extracellular vesicles [60].

The establishment of a reverberating proinflammatory and profibrotic circuit in AF revolves around inflammasome activation, especially NOD-, LRR-, and pyrin domain-containing protein (NLRP)3 [62,63]. Briefly, NLRP3 activation consists of a two-step process, namely a priming phase responsible for transcriptional upregulation of NLRP3, and an activation phase, during which NLRP3 interacts with the adaptor protein ASC (apoptosis-associated speck-like protein containing a caspase recruitment domain) and pro-caspase-1 to form the inflammasome complex. Subsequently, activated caspase 1 proteolytically cleaves pro IL-1β and pro IL-18 into their active forms, IL-1β and IL-18, which drive many events associated to AF [64].

How does PD-dependent systemic inflammation or bacteremia activate the inflammasome? It has been reported that increased cytosolic Ca^2+^ concentrations play a role in the activation of NLRP3 complex [65]. Given that PD-derived inflammatory mediators alter Ca^2+^ handling in cardiomyocyte, we suggest that the reduced diastolic Ca^2+^ reuptake into the sarcoplasmic reticulum contributes to NRLP3 activation.

Elevated cytosolic Ca^2+^ concentrations have been associated with increased mitochondrial Ca^2+^ uptake, which in turn leads to increased ROS generation and NLRP3 assembly and function [66,67]. Notably, oxidative stress has been identified as a biomarker of AF [58]. Considering that both host immunity activation and sustained inflammation result in overproduction of ROS, it is plausible that PD-driven oxidative stress also contributes to NLRP3 activation in cardiomyocytes, fibroblasts, and immune cells.

In addition to systemic pro-inflammatory mediators, circulating bacteria and their byproducts could also contribute to the activation of NLRP3 inflammasome in both immune and non-immune cells.

The cascade involving systemic inflammation/circulating bacteria/NLRP3 activation culminates in the metabolic rearrangement of immune cells, fibroblasts, and cardiomyocytes. The increased production of IL-1β, IL-18, TNF-α, and IL-6 has been associated with a further increase in NLRP3 formation and the activation of several pathways involved in atrial remodeling [62,68].

Obviously, in addition to mechanisms involving NLRP3 activation, several NLRP3-independent pathways are activated in immune cells, fibroblasts, and cardiomyocytes in response to PD-dependent systemic alterations that lead to the onset of AF [62].

Overall, a reverberating proinflammatory and profibrotic circuit was established (Figure 3). PD-dependent systemic inflammation and bacteremia activate a profibrotic phenotype in fibroblasts, while immune cells activation accounts for the release of proinflammatory cytokines. The increased production of cytokines stimulates further infiltration of activated memory CD4+ and γδ T cells and a concomitant reduction in CD8+ and Treg within atrial tissue. Higher cytokines levels also support the profibrotic state of fibroblast. Cardiomyocytes respond to both systemic mediators and local inflammation, displaying electrical disturbances. Altogether, atrial structural remodeling, fibrosis, and electrophysiological disturbances predispose to AF.

Future research should establish causal relationships between the translocation of oral pathogens and/or inflammatory mediators and the development of AF and identify the linking mechanisms. However, the role of oral microbial factors highlights the importance of maintaining periodontal health as a potentially modifiable factor in AF risk.

### 5.3. Molecular Basis of AF Development

Numerous studies have been aimed at understanding the mechanisms that lead to the onset of AF and its progression. From these studies it clearly emerged that once developed, AF can promote its own maintenance through the establishment of reverberating proinflammatory and profibrotic circuits, therefore displaying a progressive nature.

Ectopic atrial triggers and a reentry-prone substrate (modulated by tissue fibrosis and connexin abnormalities) have been recognized as key factors in the onset of AF.

Atrial ectopia occurs very commonly in the pulmonary veins, due to distinct electrophysiological properties of resident cardiomyocytes, the anatomy of the pulmonary veins, and the proximity to the major ganglia of the autonomic nervous system [69,70,71]. Mechanisms involved in atrial ectopia include Ca^2+^ handling abnormalities and oxidative stress.

The major link between Ca^2+^-dysregulation and arrhythmogenesis is the multifunctional Ca^2+^/calmodulin-dependent protein kinase type-II (CaMKII), which act synergistically with β-adrenergic receptor (βAR)/cAMP/PKA signaling pathway in promoting AF [72]. Accordingly, sympathetic stimulation sustains CaMKII activation by increasing its phosphorylation and preventing dephosphorylation of CaMKII-Thr287 [73,74].

Oxidative stress also contributes to Ca^2+^-handling abnormalities. Indeed, oxidative stress activates the stress-response kinase c-Jun N-terminal kinase-2 (JNK2), which upregulates CaMKII expression and activation [75,76]. Furthermore, during AF progression, atrial arrhythmia induces oxidative stress via CD44/NOX4 signaling, which in turn activates CaMKII through oxidation at Met281/282 [77]. Therefore, CaMKII overexpression and hyperactivation may contribute to or promote AF [78].

TGF-β signaling also contribute to AF onset and progression through different mechanisms [79]. The activation of TGF-β1/Smad2/3 pathway triggers transcription of specific genes mediating fibroblast senescence, activation of myofibroblasts, matrix remodeling, mitochondrial dysfunction, and epithelial-mesenchymal transition [80,81]. TGF-β signaling also increases NOX4 expression and activity [82]. Therefore, TGF-β signaling could contribute to AF onset by increasing fibrosis, ROS production, and Ca^2+^ handling abnormalities [59].

Recent evidence points to NLRP3-inflammasome signaling as a central mediator of the complex pathogenesis of AF [83]. Altered ion channel expression, increased cytosolic Ca^2+^ concentrations, and ROS production have been associated with NLRP3 activation in cardiomyocytes. However, the exact role of NLRP3 in atrial ectopia has not yet been established.

Conduction abnormalities are the other pathognomonic sign of AF [84]. Structural integrity of atrial tissue and effective cell-to-cell coupling, which are the main determinants of conduction, are impaired in AF. Atrial structural remodeling occurs after fibroblast activation through the stimulation of several receptors, including that for Ang-II, PDGF, and TGF-β. Upon activation, many different ECM components or enzymes, such as collagen and MMPs, respectively, are secreted and contribute to tissue remodeling. Both ROS and NLRP3, recognized as key mediators of PD-induced systemic inflammation, play a role in fibroblast activation [64,65]. Similarly, cytoplasmic Ca^2+^ could also regulate fibroblast activation [65,66].

Overall, many signals could contribute to profibrotic signaling in fibroblasts. In atrial tissue, Ang-II receptors, PDGF receptors, TGF-β receptors, ROS, NLRP3, and TRP channels act as main drivers [85,86,87]. Furthermore, mediators of systemic inflammation such as IL-6, IL-1β, TNF-α, and CCL2 have been shown to directly induce tissue remodeling [88].

Effective cell-to-cell communication at the intercalated disks is ensured by the correct assembly of connexins. Connexins form hexameric connexons permeable to small molecules, acting as hemichannels. To ensure direct cell communication, connexons from each cell align across the space between them to form a complete intercellular channel. This allows for the direct passage of molecules from one cell to another. In the atria, the principal connexins are connexin-43 (Cx43) and connexin-40 (Cx40) [89]. Many studies showed that alterations in connexins expression, assembly, and functioning increase AF-vulnerability [90]. Interestingly, systemic inflammation could also alter connexin expression, so contributing to electrical remodeling [91]. This evidence seems to suggest that PD-dependent systemic inflammation might contribute to electrical remodeling by direct altering the expression of connexins.

A correct connexins phosphorylation is required for membrane expression, specific localization, and proper functioning. Kinases involved in connexin phosphorylation include PKA, PKC, CaMKII, and MAPKs [92]. Furthermore, intracellular ROS interferes with connexin function, either directly or via the activation of kinases like CaMKII or JNK [92].

This evidence provides important considerations. CaMKII overactivation contributes to AF onset via direct alterations of Ca^2+^ handling and connexins dysfunction. Similarly, autonomic nervous system contributes to both Ca^2+^ handling abnormalities and connexins dysfunction via β-adrenergic receptor (βAR)/cAMP/PKA signaling pathway. Finally, oxidative stress also contributes through the efforts of JNK/CaMKII cascade.

While molecular mechanisms underlying AF are heavily studied, significant gaps persist, above all in defining the complex and heterogeneous pathways that drive AF. The identification of several triggers, sometimes within the same patients, makes the treatment very difficult. Similarly, although it is clear that PD-dependent systemic inflammation is a risk factor for the development of AF, there is still no evidence of a cause-and-effect relationship between PD and AF. Even less well understood are the molecular mechanisms that drive the progression from paroxysmal (intermittent) to persistent AF.

## 6. Inflammation as a Common Pathophysiological Bridge: Speculation or Experimental Evidence?

To bridge the gap between mechanistic understanding and population-level observations, clinical and genetic studies offer critical translational evidence. Large-scale epidemiological investigations have moved beyond simple associations to provide robust data on the magnitude of risk and its preventative reduction. For example, a nationwide cohort study involving 161,286 subjects demonstrated that improved oral hygiene indicators, such as frequent tooth brushing, were associated with lower risk of AF occurrence [1]; similarly, other studies showed that professional dental cleanings, as well as regular brushing, significantly reduce AF incidence [4,10]. The accumulating body of research consistently supports a compelling link between PD and AF, mediated by four key pathways: systemic inflammation (elevated CRP, IL-6), immune dysregulation, microbial translocation, and atrial remodeling. Specifically, chronic periodontal infection acts as a persistent reservoir of inflammatory mediators; meta-analyses have consistently confirmed that PD patients exhibit significantly elevated serum levels of CRP compared to healthy controls, providing the substrate for systemic inflammation known to alter atrial electrophysiology [3,4]. Most importantly, direct microbial invasion is no longer hypothetical; a landmark study recently utilized 16S rRNA gene sequencing to detect DNA signatures of *P. gingivalis* within the atrial appendages of AF patients and, crucially, demonstrated with in vivo murine models that such translocation actively drives atrial fibrosis through the specific upregulation of the Galectin-3 and TGF-β1 signaling pathways [5]. The above findings suggest that periodontal disease is not merely associated with, but may actively contribute to, the development and progression of AF via structural and electrophysiological alterations. Notably, untreated PD has been identified as an independent predictor of arrhythmia recurrence following catheter ablation for paroxysmal AF (HR 2.06), underscoring the prognostic value of periodontal status [19]. However, despite these converging lines of evidence, some critical questions remain unanswered, and we are still far from defining a causal relationship. From a clinical perspective, the above insights require stronger interdisciplinary collaboration among cardiologists, dentists, periodontists, and immunologists. Integrating oral health assessments into cardiovascular risk screening, along with patient education on the systemic benefits of oral hygiene, could represent cost-effective, preventive approaches to reducing AF burden. Multidisciplinary care teams are uniquely positioned to design and implement holistic management strategies that address both periodontal and cardiac health.

## 7. Limitations

The limitations of this study include a high heterogeneity among studies; a lack of randomized controlled trials, considered the “gold standard” for minimizing bias and demonstrating a cause–effect relationship; no evidence of causality between PD and AF; and the potential presence of residual confounding factors, including cases of undiagnosed apical PD in both controls and the PD group. The last point is crucial, as periapical dental abscess has been recognized as an independent predictor for new-onset AF (HR, 1.11, 95% CI, 1.01–1.22) [93].

## 8. Conclusions and Future Perspectives

In summary, advancing our understanding of the PD–AF interplay requires an integrated effort across basic science, clinical research, and public health. Such a comprehensive approach holds the promises of not only improving outcomes for patients affected by these common chronic conditions, but also opening new frontiers in cardiovascular prevention and precision medicine. The convergence of clinical, genetic, and population-based evidence underscores the biological plausibility of the discussed association and highlights the substantial impact that future interventional research could yield.

Future research must focus on delineating the precise biological pathways through which chronic oral inflammation could influence cardiac electrophysiology and structural remodeling. Large-scale, prospective, and interventional studies—particularly randomized controlled trials that incorporate detailed periodontal assessments and cardiovascular endpoints—are essential to establish causality and evaluate whether periodontal treatment can reduce AF incidence or recurrence, as recently proposed by the authors of [94].

Moreover, the current heterogeneity in study populations, inflammatory markers, and diagnostic criteria underscores the need for standardized research protocols. The identification of reliable biomarkers—such as circulating microRNAs, extracellular vesicles, and immunogenetic profiles—will be key to refining risk stratification and guiding personalized preventive strategies.

## Data Availability

No new data were created or analyzed in this study. Data sharing is not applicable to this article.

## Abbreviations

The following abbreviations are used in this manuscript:

AF	Atrial Fibrillation
ASC	apoptosis-associated speck-like protein containing a caspase recruitment domain
PD	Periodontitis
BOP	Bleeding On Probing
βAR	β-adrenergic receptor
CaMKII	Ca^2+^/calmodulin-dependent protein kinase type-II
cAMP	cyclic adenosine monophosphate
CCL2	C-C Motif Chemokine Ligand 2
CD40L	Cluster of Differentiation 40 ligand
CI	Confidence Interval
COPD	Chronic Obstructive Pulmonary Disease
CRP	C-Reactive Protein
Cx43	Connexin-43
Cx40	Connexin-40
ECM	Extracellular Matrix
HCN	Hyperpolarization-activated Cyclic Nucleotide-gated (channel)
HR	Hazard Ratio
HSP60/65	Heat Shock Protein 60/65
IL-1β	Interleukin-1β
IL-17	Interleukin-17
IL-18	Interleukin-18
IL-2	Interleukin-2
IL-23	Interleukin-23
IL-6	Interleukin-6
IL-8	Interleukin-8
JNK2	c-Jun N-terminal kinase-2
LAAT	Left Atrial Appendage Thrombus
LPS	Lipopolysaccharide
MAPKs	Mitogen-Activated Protein Kinases
MCP-1	Monocyte Chemoattractant Protein-1
MMP-9	Matrix Metalloproteinase-9
MMPs	Matrix Metalloproteinase
MPO	Myeloperoxidase
NLRP3	NOD-, LRR-, and pyrin domain-containing protein 3
NOX4	NADPH Oxidase 4
OR	Odds Ratio
PAI-1	Plasminogen Activator Inhibitor-1
PDGF	Platelet-Derived Growth Factor
PGE2	Prostaglandin E2
PISA	Periodontal Inflamed Surface Area
PKA	Protein Kinasi A
PKC	Protein Kinasi C
PPD	Probing Pocket Depth
RGS1	Regulator of G-protein Signaling 1
ROS	Reactive Oxygen Species
SAA	Serum Amyloid A
Smad2/3	SMAD Family Member 2/3
TIMPs	Tissue Inhibitors of Metalloproteinases
TGF-β	Transforming Growth Factor-beta
TNF-α	Tumor Necrosis Factor-α
TRP	Transient Receptor Potential
